# Supplementary material for: Extinction risk modeling predicts range-wide differences of climate change impact on Karner blue butterfly (Lycaeides melissa samuelis)
Source: PLoS One. 2023 Nov 7;18(11):e0262382. doi: 10.1371/journal.pone.0262382 (PMC10629659; doi:10.1371/journal.pone.0262382)
Supplement: S1 Table — The state, county, latitude, and longitude of each site are listed. (DOCX) [file pone.0262382.s001.docx]

# **Supporting Information**

**S1 Table. Detailed locations of 48 Kbb sampled sites of five populations.** The states, counties, latitudes, and longitudes are listed**.**

| Central Wisconsin | | | | | | | | | | | |
| --- | --- | --- | --- | --- | --- | --- | --- | --- | --- | --- | --- |
| Site | County | Latitude | Longitude | Site | County | Latitude | Longitude | Site | County | Latitude | Longitude |
| Dike 17 | WI Jackson | 44°18'36" | 90°33'50" | StanM | WI Jackson | 44°13'55" | 90°39'21" | WildSp | WI Jackson | 44°16'40" | 90°40'40" |
| Lichtner | WI Jackson | 44°22'55" | 90°41'22" | NBRE | WI Jackson | 44°18'43" | 90°44'24" | SBRW | WI Jackson | 44°17'31" | 90°44'34" |
| Bauer Cut | WI Jackson | 44°17'49" | 90°45'7" | WCM | WI Jackson | 44°16'22" | 90°45'50" | WoodCFX | WI Wood | 44°19'39" | 90°4'49" |
| CTHX | WI Wood | 44°20'24" | 90°7'48" | XS | WI Wood | 44°19'1" | 90°7'40" | XEW | WI Wood | 44°18'18" | 90°7'44" |
| Sand 5 | WI Wood | 44°19'1" | 90°11'9" | Buena | WI Portage | 44°21'36" | 89°32'60" |  |  |  |  |
| Fort McCoy, Wisconsin | | | | | | | | | | | |
| Site | County | Latitude | Longitude | Site | County | Latitude | Longitude | Site | County | Latitude | Longitude |
| A1 | WI Monroe | 43°56'5" | 90°45'6" | A5 | WI Monroe | 43°55'47" | 90°38'26" | B7 | WI Monroe | 43°57'22" | 90°38'26" |
| B8 | WI Monroe | 43°56'45" | 90°36'43" | B13 | WI Monroe | 43°58'7" | 90°42'1" | B16 | WI Monroe | 43°57'33" | 90°38'51" |
| B18 | WI Monroe | 43°58'37" | 90°40'41" | C11 | WI Monroe | 44°5'50" | 90°38'59" | D4 | WI Monroe | 44°7'11" | 90°41'16" |
| D6 | WI Monroe | 44°7'26" | 90°38'51" | E131 | WI Monroe | 44°6'50" | 90°41'56" | E132 | WI Monroe | 44°6'48" | 90°42'26" |
| Northwestern Wisconsin | | | | | | | | | | | |
| Site | County | Latitude | Longitude | Site | County | Latitude | Longitude | Site | County | Latitude | Longitude |
| BuPeet | WI Burnett | 45°54'18" | 92°32'34" | CrCorner | WI Burnett | 45°54'18" | 92°32'60" | CrReedE | WI Burnett | 45°43'7" | 92°35'13" |
| CrNRefR | WI Burnett | 45°53'13" | 92°35'60" | CrKJM | WI Burnett | 45°52'37" | 92°33'9" | Crover | WI Burnett | 45°52'40" | 92°37'55" |
| CrPhant | WI Burnett | 45°50'15" | 92°40'12" | St | WI Burnett | 45°44'6" | 92°44'24" |  |  |  |  |
| Indiana Dunes National Park | | | | | | | | | | | |
| Site | County | Latitude | Longitude | Site | County | Latitude | Longitude | Site | County | Latitude | Longitude |
| Miller | IN Porter | 41°36'29" | 87°16'43" | Tolleston | IN Porter | 41°36'23" | 87°15'19" | Marquette | IN Porter | 41°36'55" | 87°13'9" |
| Long Lake | IN Porter | 41°36'52" | 87°12'59" | Inland | IN Porter | 41°36'55" | 87°12'36" | West Beach | IN Porter | 41°37'5" | 87°12'51" |
| Albany Pine Bush, New York | | | | | | | | | | | |
| Site | County | Latitude | Longitude | Site | County | Latitude | Longitude | Site | County | Latitude | Longitude |
| PBNW | NW Albany | 42°44'39" | 73°54'8" | BH | NW Albany | 42°44'14" | 73°53'10" | KRBE | NW Albany | 42°43'19" | 73°52'0" |
| KBE | NW Albany | 42°44'56" | 73°51'39" | KBW | NW Albany | 42°43'0" | 73°52'55" | PBSW | NW Albany | 42°42'27" | 73°53'49" |
| PBSE | NW Albany | 42°41'52" | 73°51'55" | KRBE | NW Albany | 42°43'34" | 73°52'39" |  |  |  |  |
