# Supplementary material for: Extinction risk modeling predicts range-wide differences of climate change impact on Karner blue butterfly (Lycaeides melissa samuelis)
Source: PLoS One. 2023 Nov 7;18(11):e0262382. doi: 10.1371/journal.pone.0262382 (PMC10629659; doi:10.1371/journal.pone.0262382)
Supplement: S1 File — (PDF) [file pone.0262382.s004.pdf]

# Supporting Information

~~~~
```{r}
library(car)
library(lattice)
library(caret)
library(mgcv)
library(nlme)
library(leaps)
library(glmbb)
library(pls)
library(mdatools)
library(gbm)
library(randomForest)
library(rfUtilities)
library(nnet)
library(kernlab)
library(e1071)
library(earth)
library(biomod2)
library(MASS)
```
~~~~

~~~~
```{r}
Twenty_Two_Sites = read.csv("C:\\Users\\leebo\\Documents\\R  TIme Wilder\\Weighted-OnetoTwo-Spatial.csv")
```
~~~~

~~~~
```{r}
~~~~

````r
SSCA = scale(Twenty_Two_Sites$TTCA)
SSCG = scale(Twenty_Two_Sites$TTCG)
SSCS = scale(Twenty_Two_Sites$TTCS)
SSCF = scale(Twenty_Two_Sites$TTCF)
SSCMT_AG  = scale(Twenty_Two_Sites$MT_AG)
SSCMT_JL = scale(Twenty_Two_Sites$MT_JL)
SSCMT_JN = scale(Twenty_Two_Sites$MT_JN)
SSCPT_AG = scale(Twenty_Two_Sites$PP_AG)
SSCPT_JL = scale(Twenty_Two_Sites$PP_JL)
SSCPT_JN = scale(Twenty_Two_Sites$PP_JN)
SSCIT_AG = scale(Twenty_Two_Sites$IT_AG)
SSCIT_JL = scale(Twenty_Two_Sites$IT_JL)
SSCIT_JN = scale(Twenty_Two_Sites$IT_JN)
SSCAT_AG  = scale(Twenty_Two_Sites$AT_AG)
SSCAT_JL = scale(Twenty_Two_Sites$AT_JL)
SSCAT_JN = scale(Twenty_Two_Sites$AT_JN)
Location = Twenty_Two_Sites$Location
Longitude = Twenty_Two_Sites$Longitude
Latitude = Twenty_Two_Sites$Latitude
Slope = Twenty_Two_Sites$Slope
Trasp = Twenty_Two_Sites$Trasp
Elevation = Twenty_Two_Sites$Elevation
Canopy = Twenty_Two_Sites$Canopy
```
````

````
```{r}
````

````
lm_SSC = lm(SSCA ~ (SSCG + SSCS + SSCF) + (SSCMT_AG + SSCMT_JL + SSCMT_JN + SSCPT_AG + SSCPT_JL + SSCPT_JN + SSCIT_AG + SSCIT_JL + SSCIT_JN + SSCAT_AG + SSCAT_JL + SSCAT_JN) + (Elevation + Slope + Trasp + Canopy) + I(Longitude * Latitude))

```
````

````
```{r}

summary(lm_SSC)

plot(lm_SSC)

hist(SSCA)

boxcox(lm_SSC)

```
````

````
```{r}

df_SSC = data.frame(SSCA = SSCA, SSCG = SSCG, SSCS = SSCS, SSCF = SSCF, SSCMT_AG = SSCMT_AG, SSCMT_JL = SSCMT_JL, SSCMT_JN = SSCMT_JN, SSCPT_AG = SSCPT_AG, SSCPT_JL = SSCPT_JL, SSCPT_JN = SSCPT_JN, SSCIT_AG = SSCIT_AG, SSCIT_JL = SSCIT_JL, SSCIT_JN = SSCIT_JN, SSCAT_AG = SSCAT_AG, SSCAT_JL = SSCAT_JL, SSCAT_JN = SSCAT_JN, Longitude = Longitude, Latitude = Latitude, Slope = Slope, Trasp = Trasp, Elevation = Elevation, Canopy = Canopy)

```
````

````
```{r}

multi.collinear(df_SSC)

```
````

````
```{r}

fitControl_SSC = trainControl(method = "repeatedcv", number = 5, repeats = 5)

```
````

````
```{r}
````

```
set.seed(7)

caret_lr_SSC = train(SSCA ~ (SSCG + SSCS + SSCF) + (SSCMT_AG + SSCMT_JL + SSCMT_JN + SSCPT_AG + SSCPT_JL + SSCPT_JN + SSCIT_AG + SSCIT_JL + SSCIT_JN + SSCAT_AG + SSCAT_JL + SSCAT_JN) + (Elevation + Slope + Trasp + Canopy) + I(Longitude * Latitude), data = df_SSC, method = "leapSeq", trControl = fitControl_SSC)

print(caret_lr_SSC)

varImp_lr_SSC = varImp(object = caret_lr_SSC)

print(varImp_lr_SSC)

plot(varImp_lr_SSC, main = "LR")
```

````
```{r}

set.seed(7)

caret_glm_SSC = train(SSCA ~ (SSCG + SSCS + SSCF) + (SSCMT_AG + SSCMT_JL + SSCMT_JN + SSCPT_AG + SSCPT_JL + SSCPT_JN + SSCIT_AG + SSCIT_JL + SSCIT_JN + SSCAT_AG + SSCAT_JL + SSCAT_JN) + (Elevation + Slope + Trasp + Canopy) + I(Longitude * Latitude), data = df_SSC, method = "glm", trControl = fitControl_SSC)

print(caret_glm_SSC)

varImp_glm_SSC = varImp(object = caret_glm_SSC)

print(varImp_glm_SSC)

plot(varImp_glm_SSC, main = "GLM")

```
````

````
```{r}

set.seed(7)

caret_pls_SSC = train(SSCA ~ (SSCG + SSCS + SSCF) + (SSCMT_AG + SSCMT_JL + SSCMT_JN + SSCPT_AG + SSCPT_JL + SSCPT_JN + SSCIT_AG + SSCIT_JL + SSCIT_JN + SSCAT_AG + SSCAT_JL + SSCAT_JN) + (Elevation + Slope + Trasp + Canopy) + I(Longitude * Latitude), data = df_SSC, method = "kernelpls", trControl = fitControl_SSC)

print(caret_pls_SSC)

varImp_pls_SSC = varImp(object = caret_pls_SSC)

print(varImp_pls_SSC)
````
````

````
plot(varImp_pls_SSC,  main = "PLS")

```
````

````
```{r}

set.seed(7)

caret_gbm_SSC = train(SSCA ~ (SSCG + SSCS + SSCF) + (SSCMT_AG  + SSCMT_JL + SSCMT_JN + SSCPT_AG + SSCPT_JL + SSCPT_JN + SSCIT_AG + SSCIT_JL + SSCIT_JN + SSCAT_AG  + SSCAT_JL + SSCAT_JN) + (Elevation + Slope + Trasp + Canopy) + I(Longitude * Latitude), data = df_SSC, method = "gbm", trControl = fitControl_SSC)

print(caret_gbm_SSC)

```{r}

varImp_gbm_SSC  = varImp(object = caret_gbm_SSC)

print(varImp_gbm_SSC)

plot(varImp_gbm_SSC,  main = "GBM")

```

```
````

````
```{r}

set.seed(7)

caret_rf_SSC = train(SSCA  ~ (SSCG + SSCS + SSCF) + (SSCMT_AG  + SSCMT_JL + SSCMT_JN + SSCPT_AG + SSCPT_JL + SSCPT_JN + SSCIT_AG + SSCIT_JL + SSCIT_JN + SSCAT_AG + SSCAT_JL + SSCAT_JN) + (Elevation + Slope + Trasp + Canopy) + I(Longitude * Latitude), data = df_SSC, method = "rf", importance = TRUE, trControl = fitControl_SSC)

print(caret_rf_SSC)

varImp_rf_SSC = varImp(object = caret_rf_SSC)

print(varImp_rf_SSC)

plot(varImp_rf_SSC,  main = "RF")

```
````

````
```{r}
````

~~~
set.seed(7)

caret_ann_SSC = train(SSCA  ~ (SSCG + SSCS + SSCF) + (SSCMT_AG  + SSCMT_JL + SSCMT_JN + SSCPT_AG + SSCPT_JL + SSCPT_JN + SSCIT_AG + SSCIT_JL + SSCIT_JN + SSCAT_AG + SSCAT_JL + SSCAT_JN) + (Elevation + Slope + Trasp + Canopy) + I(Longitude * Latitude), data = df_SSC, method = "avNNet", trControl = fitControl_SSC)

print(caret_ann_SSC)

varImp_ann_SSC  = varImp(object = caret_ann_SSC)

print(varImp_ann_SSC)

plot(varImp_ann_SSC,  main = "ANN")
```
~~~

~~~
```{r}
set.seed(7)

caret_svm_SSC  = train(SSCA  ~ (SSCG + SSCS + SSCF) + (SSCMT_AG  + SSCMT_JL + SSCMT_JN + SSCPT_AG + SSCPT_JL + SSCPT_JN + SSCIT_AG + SSCIT_JL + SSCIT_JN + SSCAT_AG + SSCAT_JL + SSCAT_JN) + (Elevation + Slope + Trasp + Canopy) + I(Longitude * Latitude), data = df_SSC, method = "svmRadial", trControl = fitControl_SSC)

print(caret_svm_SSC)

varImp_svm_SSC  = varImp(object = caret_svm_SSC)

print(varImp_svm_SSC)

plot(varImp_svm_SSC,  main = "SVM")
```
~~~

~~~
```{r}
set.seed(7)

caret_mars_SSC  = train(SSCA  ~ (SSCG + SSCS + SSCF) + (SSCMT_AG  + SSCMT_JL + SSCMT_JN  + SSCPT_AG + SSCPT_JL + SSCPT_JN + SSCIT_AG + SSCIT_JL + SSCIT_JN + SSCAT_AG  + SSCAT_JL + SSCAT_JN) + (Elevation + Slope + Trasp + Canopy) + I(Longitude  * Latitude),   data = df_SSC, method = "bagEarth", trControl = fitControl_SSC)

print(caret_mars_SSC)

varImp_mars_SSC  = varImp(object = caret_mars_SSC)

print(varImp_mars_SSC)
~~~

````
plot(varImp_mars_SSC,  main = "MARS")
```
````

````
```{r}
comparisons_SSC  = resamples(list(LR  = caret_lr_SSC, GLM = caret_glm_SSC, PLS = caret_pls_SSC, GBM = caret_gbm_SSC, RF = caret_rf_SSC, ANN = caret_ann_SSC, SVM = caret_svm_SSC, MARS = caret_mars_SSC))
summary(comparisons_SSC)
bwplot(comparisons_SSC)
dotplot(comparisons_SSC)
```
````

````
```{r}
````

```
set.seed(7)

rfeControl_SSC_rf = rfeControl(functions = rfFuncs, method = "repeatedcv", number = 5, repeats = 5)

rfe_SSC_rf = rfe(df_SSC, SSCA, sizes = c(1:21), rfeControl = rfeControl_SSC_rf)

```
```

```
```{r}

print(rfe_SSC_rf)

predictors(rfe_SSC_rf)

plot(rfe_SSC_rf, type=c("g", "o"))

```
```

```
```{r}

set.seed(7)

gaControl_SSC_rf = gafsControl(functions = rfGA, method = "repeatedcv", number = 5, repeats = 5)

ga_SSC_rf <- gafs(dfp_SSC, SSCA, iters = 5, gafsControl = gaControl_SSC_rf)

print(ga_SSC_rf)

plot(ga_SSC_rf, type=c("g", "o"))

print(ga_SSC_rf$ga$final)

```
```

```
```{r}

set.seed(7)

saControl_SSC_rf = safsControl(functions = rfSA, method = "repeatedcv", number = 5, repeats = 5)

sa_SSC_rf = safs(df_SSC, SSCA, iters = 5, safsControl = saControl_SSC_rf)

print(sa_SSC_rf)

plot(sa_SSC_rf, type=c("g", "o"))

print(sa_SSC_rf$sa$final)

```

```{r}
```

```
rfu_SSC = rf.modelSel(df_SSC, SSCA, seed = 7, imp.scale = "se")

print(rfu_SSC)

plot(rfu_SSC)

```

```
```{r}

set.seed(7)

caret_rf_SSC_rfe = train(SSCA ~ (SSCG + SSCS + SSCF) + (SSCPT_AG + SSCPT_JN + SSCAT_AG + SSCAT_JL), data = df_SSC, method = "rf", importance = TRUE, trControl = fitControl_SSC)


caret_rf_SSC_ga = train(SSCA ~ (SSCG + SSCS + SSCF) + (SSCMT_JL + SSCPT_AG + SSCPT_JN + SSCAT_JL + SSCAT_AG), data = df_SSC, method = "rf", importance = TRUE, trControl = fitControl_SSC)


caret_rf_SSC_sa = train(SSCA ~ (SSCMT_JN + SSCPT_JL + SSCIT_JL + SSCIT_AG) + (Slope + Trasp + Elevation), data = df_SSC, method = "rf", importance = TRUE, trControl = fitControl_SSC)


caret_rf_SSC_rfu = train(SSCA ~ (SSCS + SSCF) + (SSCPT_JN + SSCPT_AG + SSCIT_JN), data = df_SSC, method = "rf", importance = TRUE, trControl = fitControl_SSC)

```
```

```
```{r}

comparisons_SSC_RF = resamples(list(RFE = caret_rf_SSC_rfe, GA = caret_rf_SSC_ga, SA = caret_rf_SSC_sa, RFU = caret_rf_SSC_rfu))

summary(comparisons_SSC_RF)

bwplot(comparisons_SSC_RF)

dotplot(comparisons_SSC_RF)

```
```

```
```{r}
```
```

````
varImp_rf_SSC_rfe = varImp(object = caret_rf_SSC_rfe)

print(varImp_rf_SSC_rfe)

plot(varImp_rf_SSC_rfe, main = "RF")

```
````

````
```{r}

varImp_rf_SSC_ga = varImp(object = caret_rf_SSC_ga)

print(varImp_rf_SSC_ga)

plot(varImp_rf_SSC_ga, main = "RF")

```
````

````
```{r}

varImp_rf_SSC_sa = varImp(object = caret_rf_SSC_sa)

print(varImp_rf_SSC_sa)

plot(varImp_rf_SSC_sa, main = "RF")

```
````

````
```{r}

varImp_rf_SSC_rfu = varImp(object = caret_rf_SSC_rfu)

print(varImp_rf_SSC_rfu)

plot(varImp_rf_SSC_rfu, main = "RF")

```
````

````
```{r}
````

````
pls_SSC_cv5 = pls(df_SSC, SSCA, scale = TRUE, cv = 5, ncomp.selcrit = "min")

summary(pls_SSC_cv5)

```
````

````
```{r}

pls_SSC_cv5 = pls(dfp_SSC, SSCA, ncomp = 5, scale = TRUE, cv = 5, ncomp.selcrit = "min")

plotVIPScores(pls_SSC_cv5,  type = 'h', show.labels = T)

plotSelectivityRatio(pls_SSC_cv5,  type = 'b', show.labels = T)

plotSelectivityRatio(pls_SSC_cv5,  ncomp = 5, type = 'h', show.labels = T)

```
````

````
```{r}

excl_cv5_VIP0.5 = getVIPScores(pls_SSC_cv5,  ncomp = 5) < 0.5

show(excl_cv5_VIP0.5)

pls_SSC_cv5_VIP0.5  = pls(dfp_SSC,  SSCA, scale = T, cv = 5, exclcols = excl_cv5_VIP0.5)

summary(pls_SSC_cv5_VIP0.5)

```
````

````
```{r}

excl_cv5_SR0.1 = getSelectivityRatio(pls_SSC_cv5,  ncomp = 5) < 0.1

show(excl_cv5_SR0.1)

pls_SSC_cv5_SR0.1  = pls(dfp_SSC,  SSCA, scale = T, cv = 5, exclcols = excl_cv5_SR0.1)

summary(pls_SSC_cv5_SR0.1)

```
````

````
```{r}
````

```
plsjk_SSC_cv5 = pls(dfp_SSC, SSCA, scale = TRUE, cv = 5, ncomp = 5, coeffs.ci = 'jk', ncomp.selcrit = "min")

plotRegcoeffs(plsjk_SSC_cv5, type = 'h', show.labels = T)

plotRegcoeffs(plsjk_SSC_cv5, type = 'h', show.labels = T, show.ci = F)

summary(plsjk_SSC_cv5$coeffs, ncomp = 1)

```
````

````
```{r}

excl_cv5_p0.05 = plsjk_SSC_cv5$coeffs$p.values[, 2, 1] > 0.05

show(excl_cv5_p0.05)

plsjk_SSC_cv5_p0.05 = pls(dfp_SSC, SSCA, scale = T, cv = 5, exclcols = excl_cv5_p0.05)

summary(plsjk_SSC_cv5_p0.05)

```
````

````
```{r}

set.seed(7)

caret_plsjk_SSC = train(SSCA ~ (SSCG + SSCS + SSCF) + (SSCMT_AG + SSCMT_JL + SSCPT_AG + SSCIT_JL + SSCAT_AG + SSCAT_JL), data = df_SSC, method = "kernelpls", trControl = fitControl_SSC)

print(caret_plsjk_SSC)

varImp_plsjk_SSC = varImp(object = caret_plsjk_SSC)

print(varImp_plsjk_SSC)

plot(varImp_plsjk_SSC, main = "PLS")

```
````

````
```{r}

set.seed(7)

caret_rf_SSC_pls = train(SSCA ~ (SSCG + SSCS + SSCF) + (SSCMT_AG + SSCMT_JL + SSCPT_AG + SSCIT_JL + SSCAT_AG + SSCAT_JL), data = df_SSC, method = "rf", importance = TRUE, trControl = fitControl_SSC)

```

```{r}
````

````
comparisons_SSC_RF_PLS  = resamples(list(RF  = caret_rf_SSC_ga, PLS = caret_plsjk_SSC,  RF_PLS = caret_rf_SSC_pls))

summary(comparisons_SSC_RF_PLS)

bwplot(comparisons_SSC_RF_PLS)

dotplot(comparisons_SSC_RF_PLS)

```
````

````
```{r}

set.seed(7)

caret_rf_SSC_ga_final = train(SSCA  ~ (SSCG + SSCS + SSCF) + (SSCMT_JL + SSCPT_AG  + SSCIT_JL + SSCAT_JL + SSCAT_AG),  data = df_SSC, method = "rf", importance = TRUE, trControl = fitControl_SSC)

```
````

````
```{r}

comparisons_SSC_RF_PLS  = resamples(list(RF  = caret_rf_SSC_ga, RF_FINAL = caret_rf_SSC_ga_final))

summary(comparisons_SSC_RF_PLS)

bwplot(comparisons_SSC_RF_PLS)

dotplot(comparisons_SSC_RF_PLS)

```
````

````
```{r}
````

```
rf_cor = c()

for (i in 1:100){

  rf_SSC = randomForest(SSCD  ~ SSCG + SSCS + SSCMT_OW  + SSCIT_OW + SSCPT_OW  + SSCPT_SP +
SSCAT_OW, data = df_SSC, mtry = 2)

  predicted_rf  = predict(rf_SSC,  newdata = df_SSC)

  rf_cor[i] = cor(predicted_rf,  SSCD)^2

}

mean(rf_cor)

```
```

```
```{r}

bs_cor = c()

for (i in 1:100){

  df_SSC$ID = seq(1, nrow(df_SSC),  by=1)

  df_train = df_SSC[sample(c(1:nrow(df_SSC)),  101, replace = TRUE),]

  df_test = df_SSC[!(df_SSC$ID)  %in% (df_train$ID),]

  rf_SSC_cv = randomForest(SSCD  ~ SSCG + SSCS + SSCMT_OW  + SSCIT_OW + SSCPT_OW + SSCPT_SP  +
SSCAT_OW, data = df_train, mtry = 2)

  predicted_rf  = predict(rf_SSC_cv,  newdata  = df_test)

  bs_cor[i] = cor(predicted_rf,  df_test$SSCD)^2

}

mean(bs_cor)

```
```

```
```{r}
```

```
S = read.csv("C:/Users/leebo/Documents/R  Integration/Weighted-TwotoOne-Spatial-Swengel.csv")
W = read.csv("C:/Users/leebo/Documents/R  Integration/Weighted-TwotoOne-Spatial-Wilder.csv")
C = read.csv("C:/Users/leebo/Documents/R  Integration/Weighted-TwotoOne-Spatial-Campbell.csv")
K = read.csv("C:/Users/leebo/Documents/R  Integration/Weighted-TwotoOne-Spatial-Knuston.csv")
```

```{r}
set.seed(7)
rfeControl_SSC_rf= rfeControl(functions = rfFuncs, method = "cv", number = 5)
rfe.func_DOW<-function(x, S, W, C, K, rfeControl_SSC_rf){
  require(caret)


  s.sample = S[sample(nrow(S),nrow(C)),]
  w.sample = W[sample(nrow(W),nrow(C)),]
  c.sample = W[sample(nrow(C),nrow(C)),]
  k.sample = W[sample(nrow(K),nrow(C)),]


  train.sample = rbind(s.sample,  w.sample, c.sample, k.sample)
  preds.x <- train.sample[,-c(1,2,3,25)]
  resp.y <- train.sample[,"TTCA"]


  rfe_SSC_rf <- rfe(preds.x, resp.y, sizes = c(1:21), rfeControl = rfeControl_SSC_rf)
}
```

```{r}
```

```
cl <- makeCluster(3)
registerDoParallel(cl)
system.time({
  df.Out<-foreach(x = c(1:10)) %dopar% rfe.func_DOW(x, S, W, C, K, rfeControl_SSC_rf)
})
stopCluster(cl)

preds.comb <- data.frame(preds = NA)
for(i in 1:length(df.Out)){
  preds.sel<-data.frame(preds=df.Out[[i]]$optVariables)
  preds.comb<-rbind(preds.comb,preds.sel)
}
table(preds.comb$preds)
```
```

```
```{r}
set.seed(7)
gaControl_SSC_rf = gafsControl(functions = rfGA, method = "cv", number = 5)
ga.func<-function(x, S, W, C, K, gaControl_SSC_rf){
  require(caret)
  s.sample = S[sample(nrow(S),nrow(C)),]
  w.sample = W[sample(nrow(W),nrow(C)),]
  c.sample = W[sample(nrow(C),nrow(C)),]
  k.sample = W[sample(nrow(K),nrow(C)),]

  train.sample = rbind(s.sample,  w.sample, c.sample, k.sample)
  preds.x <- train.sample[,-c(1,2,3,25)]
  resp.y <- train.sample[,"TTCA"]
```

```
  ga_SSC_rf <- gafs(preds.x, resp.y, iters = 5, gafsControl = gaControl_SSC_rf)
}
```
```
```{r}
cl <- makeCluster(3)
registerDoParallel(cl)
system.time({
  df.Out<-foreach(x = c(1:10)) %dopar% ga.func(x, S, W, C, K, gaControl_SSC_rf)
})
stopCluster(cl)

preds.comb <- data.frame(preds = NA)
for(i in 1:length(df.Out)){
  preds.sel<-data.frame(preds=df.Out[[i]]$ga$final)
  preds.comb<-rbind(preds.comb,preds.sel)
}
table(preds.comb$preds)
```
```

```
```{r}
set.seed(7)
saControl_SSC_rf = safsControl(functions = rfSA, method = "cv", number = 5)
sa.func<-function(x, S, W, C, K, saControl_SSC_rf){
  require(caret)
  s.sample = S[sample(nrow(S),nrow(C)),]
  w.sample = W[sample(nrow(W),nrow(C)),]
  c.sample = W[sample(nrow(C),nrow(C)),]
  k.sample = W[sample(nrow(K),nrow(C)),]
```
```

~~~~
  train.sample  = rbind(s.sample,  w.sample, c.sample, k.sample)

  preds.x <- train.sample[,-c(1,2,3,25)]

  resp.y <- train.sample[,"TTCA"]


  sa_SSC_rf <- safs(preds.x,  resp.y, iters = 5, safsControl = saControl_SSC_rf)

}

```
~~~~

~~~~
```{r}

cl <- makeCluster(3)

registerDoParallel(cl)

system.time({

  df.Out<-foreach(x = c(1:10)) %dopar% sa.func(x, S, W, C, K, saControl_SSC_rf)

})

stopCluster(cl)


preds.comb <- data.frame(preds  = NA)

for(i in 1:length(df.Out)){

  preds.sel<-data.frame(preds=df.Out[[i]]$sa$final)

  preds.comb<-rbind(preds.comb,preds.sel)

}

table(preds.comb$preds)

```
~~~~

~~~~
```{r}
~~~~

````
rfu.func<-function(x, S, W, C, K){
  require(rfUtilities)
  s.sample = S[sample(nrow(S),nrow(C)),]
  w.sample = W[sample(nrow(W),nrow(C)),]
  c.sample = W[sample(nrow(C),nrow(C)),]
  k.sample = W[sample(nrow(K),nrow(C)),]

  train.sample = rbind(s.sample,  w.sample, c.sample, k.sample)
  preds.x <- train.sample[,-c(1,2,3,25)]
  resp.y <- train.sample[,"TTCA"]

  rfu_SSC <- rf.modelSel(preds.x,  resp.y, seed = 1234, imp.scale = "se")
}
```
````

````
```{r}
cl <- makeCluster(3)
registerDoParallel(cl)
system.time({
  df.Out<-foreach(x = c(1:10)) %dopar% rfu.func(x, S, W, C, K)
})
stopCluster(cl)

preds.comb <- data.frame(preds = NA)
for(i in 1:length(df.Out)){
  preds.sel<-data.frame(preds=df.Out[[i]]$selvars)
  preds.comb<-rbind(preds.comb,preds.sel)


}
````

````
table(preds.comb$preds)

```
````
````
