# Supplementary material for: Extinction risk modeling predicts range-wide differences of climate change impact on Karner blue butterfly (Lycaeides melissa samuelis)
Source: PLoS One. 2023 Nov 7;18(11):e0262382. doi: 10.1371/journal.pone.0262382 (PMC10629659; doi:10.1371/journal.pone.0262382)
Supplement: Attachment — Submitted filename: PLOS_butterfly.pdf [file pone.0262382.s005.pdf]

Review of "Extinction risk modeling predicts range-wide differences of climate change impact on Karner blue butterfly "

The authors investigate the relationship between abundance measures of Karner blue butterflies and climate variables. Specifically, they look at first and second generation counts of butterflies per kilometer for each year of data across a collection of locations and relate these counts to climate variables as well as previous generation counts, to study their annual variation. In general, I find the paper well-written and the data and application interesting. However, I have some questions pertaining to the model specification and inference that can be obtained. General and specific comments are given below.

- line 21: "developing adaptive.....?" Missing a word?
- Terminology throughout implies causal inference, yet observational data and regression-based models don't imply causality.
- line 68: Would survival models be more appropriate in this analysis? How does the survival of generation impact the next?
- line 172: Is this suppose to be Table 2?
- Why fit separate models to the first and second generations? Seems like one model should be fitted with identifiers for the different generations. Indicator variables and interaction terms could be used to assess significant differences between the generations as well as their respective climate drivers.
- What distributional assumptions are being made by the model? Normal errors? Is this reasonable for this type of data (individuals per km)?
- What exactly are the data for each observation point and how are they aggregated over transects, regions, time? That is, what exactly is being grouped by ecoregion and overall population? How many observations are being used to fit the model? Just one per year? One per year per ecoregion? One per year per transect within each ecoregion?
- Why are the authors interested in the "six modeling algorithms of R package caret?" That is, why compare these off-the-shelf models? Is this just exploratory data analysis? If so, remove from the text. If not, justify why it was done and what was gained. Overall, it seems as though the authors want to fit an autoregressive model to study the relationships between Y_t and X_t as well as Y_t and Y_{t-1} (here, Y_t is the response at time t, X_t is a set of covariates associated with time t, and Y_{t-1} is the response at the previous time). If this is true, the authors should write out the model and provide discussion of the utility of each model component.

- Individual model fits (for generations and locations) prevent clear inference in terms of determining significant differences across space and between the two generations. One model that incorporates observations across all locations and generations would be much more useful. That is, stack up the response variables and predictor variables and then specify site specific and generation specific coefficients.
- line 268: How do you assess which is more influential?
- How does a model that regresses Y_t on Y_{t-1} lead to inference with regard to density dependence? Density dependence typically refers to a relationship between population growth rate and population density. If I'm understanding the model as written, you are actually just identifying positive autocorrelation.
- What is the model for Y_1, i.e., how do you model the count data from year 1?
