# Supplementary material for: Extinction risk modeling predicts range-wide differences of climate change impact on Karner blue butterfly (Lycaeides melissa samuelis)
Source: PLoS One. 2023 Nov 7;18(11):e0262382. doi: 10.1371/journal.pone.0262382 (PMC10629659; doi:10.1371/journal.pone.0262382)
Supplement: Attachment — Submitted filename: Response to Reviewers.docx [file pone.0262382.s006.docx]

# Reviewer 1:

1. **Line 21: "developing adaptive.....?" Missing a word?**

Response: Yes, " to develop adaptive management plans " is missing, and has been added back.

Revision: *(line 19-20)* Evaluating how climate shapes the population trend of the Kbb is necessary to develop adaptive management plans.

2. **Terminology throughout implies causal inference, yet observational data and regression-based models don't imply causality.**

Response: We have changed all the words of causality indicators (e.g., affect, impact, influence, cause, increase/decrease) closely related to the outputs of our models to the alternatives (e.g., associated, predict, likely, risk, probability) in Results and Discussion, except when referring to other experimental studies.

3. **Line 68: Would survival models be more appropriate in this analysis? How does the survival of generation impact the next?**

Response: Survival model is typically used for describing or predicting the survivorship or mortality of a population throughout the individuals' whole lifespan. In our study, the data we have are restricted to sampling on adult Kbbs only, instead of repeated measures on tracked individuals from egg to death. On the other hand, between-generation survival model is rare. The survival rate should range from 0 to 1. However, in our study, though there are decreasing population trends annually, the population size of the second generation is usually larger than the first generation of the same year, resulting in a "survival rate" out of this range. Therefore, we thought that both within-generation and between-generation survival models may not be suitable for our study.

4. **Line 172: Is this supposed to be Table 2?**

Response: Yes, it should be refereeing to S1 Table 2. However, we made two changes in the revised manuscript: 1. S1 Table 2 was moved to the manuscript as Table 1 because this is very important to display more details about the raw data; 2. This sentence was moved from the Statistical Analysis sub-section to the Density-Dependent Data sub-section because we wanted to clarify how we handled this count data earlier (further explained in the response to comment #7).

Revision: *(line 102-105)* Table 1

5. **Why fit separate models to the first and second generations? Seems like one model should be fitted with identifiers for the different generations. Indicator variables and interaction terms could be used to assess significant differences between the generations as well as their respective climate drivers.**

Response: The two generations occur in different periods of time in a year, mostly nonoverlapped (first generation: from fall & winter of the previous year to June in the current year: second generation: throughout the whole summer--from Jun to Aug). Since we are interested in how the adult density of each generation is affected by the climate patterns occurred within its own lifecycle, including both generations in one model would result in an increase of the degree of freedom due to more unnecessary variables involved. For example, precipitations of Jun, Jul, Aug may have direct impacts on the second generation, but the precipitations of winter and spring do not. Instead, it is the winter and spring precipitations that directly influence the first generation (e.g., survival and reproduction), then in turn affecting the second generation (all individuals are hatched from the eggs of the previous generation), which is also related to the concept of "transgenerational effect" we introduced in the manuscript. Therefore, it's more reasonable for us to include the densities of previous generations into the model rather than the climate drivers in unrelated months that only have indirect effects; otherwise, the number of variables in our models would be tripled. In addition, even if we do not separate models, the interactions between generations and monthly climate variables are not valid and meaningful, because again the second generation never occur in the winter and spring (similar for the first generation in mid-late summer). We tried to better clarify the season why we separated models to avoid confusion in the revised manuscript.

Revision 1: *(line 57-62)* As a bivoltine species, first-generation Kbbs overwinter as eggs until early April, hatch as larvae feeding on wild lupines and become pupae undergoing a metamorphosis in mid-May and emerge as adults in June with a lifespan of 5-7 days to reproduce. The second generation occurs during the summer, with hatching, pupation, and emergence in early June, late June, and early July, respectively [37]. Their thermal and drought tolerances are specific to each life stage [38-41].

Revision 2: *(line 130-132)* We modeled the first and the second generation separately because they were mostly non-overlapped except in June and experienced different climate patterns [73-74]. In total, we created 12 models: 6 populations * 2 generations.

6. **What distributional assumptions are being made by the model? Normal errors? Is this reasonable for this type of data (individuals per km)?**

Response: Both the random forest that we stuck to and the partial least square to prevent overfitting and improve interpretability makes no assumptions on the probability density of the response variable. There is no problem to handle different types of data without transformation, including skewed and discrete ones (i.e., density) in our study. Distributional assumptions do matter for the maximum-likelihood-based algorithms such as OLS, GLM, and GAM, but we decided to remove these and other machine learnings that are not random forest or partial least square from the manuscript (further explained in the response to comment #8 below).

Revision: *(line 158-159)* As a non-parametric approach, both RF and PLS make no assumptions on data distribution shape.

7. **What exactly are the data for each observation point and how are they aggregated over transects, regions, time? That is, what exactly is being grouped by ecoregion and overall population? How many observations are being used to the model? Just one per year? One per year per ecoregion? One per year per transect within each ecoregion?**

Response 1: The raw data shared with us were the counts of adult Kbb individuals per survey using one transect about 1 km length on each site (or observation point), and several surveys were made per generation (or flight period) per year. We added the total count of individuals into the new Table 1.

Response 2: The data used for fitting models were densities or derived from densities. We actually made an error on writing out the unit of density: it was individuals per ha instead of per km. Individuals per km is only an intermediate product of our calculation of density. This was corrected in the revised manuscript.

Response 3: We also decided to remove the concept of ecoregion, because these five populations occupy five different ecoregions. Therefore, it would be more straightforward to make direct inference on each population rather than ecoregion.

Response 4: We calculated the average density for each site across several transects carried out during a flight period (or generation) per year, so for the models of both generations, the number of "observations" was equal to the number of sites surveyed for a population (new Table 1) in that year (i.e., one per generation per year per site within each population).

Revision 1: *(line 94-97)* Because the number of surveys per flight period and the total length of transect per survey varied widely across researchers and years, we calculated mean counts per kilometer of transect generation-by-generation for each location. We estimated density as the number of adult individuals per hectare (ha) by assuming that a kilometer of transect represented a 2.5-ha area [64].

Revision 2: *(line 128-135)* We included both density-dependent and -independent variables to build endo-exogenous models specific to (i) each population, and (ii) all the surveyed locations of the five populations pooled together (All). We modeled the first and the second generation separately because they were mostly non-overlapped except in June and experienced different climate patterns [73-74]. In total, we created 12 models: 6 populations * 2 generations. Each model had λ as the response variable and 18 standardized explanatory variables: two previous-generation densities, four (IT, AT, MT, and PT) * three (egg, larvae/pupae, and flight) monthly climates, three topographies (elevation, slope, and trasp), and tree canopy.

8. **"Why are the authors interested in the ""six modeling algorithms of R package caret?"" That is, why compare these off-the-shelf models? Is this just exploratory data analysis? If so, remove from the text. If not, justify why it was done and what was gained. Overall, it seems as though the authors want to fit an autoregressive model to study the relationships**

**between Yt and Xt as well as Yt and Yt-1 (here, Yt is the response at time t, Xt is a set of covariates associated with time t, and Yt-1 is the response at the previous time). If this is true, the authors should write out the model and provide discussion of the utility of each model component."**

Response 1: We originally wanted to compare which would work the best for our data, but we just decided to follow your suggestion by removing the process of selecting modeling algorithms from the manuscript and stick to random forest. The SI Table 4 was thus removed.

Response 2: The model equation was included and explained in the revised manuscript. It's not autoregressive because we used λt instead of Yt (further explained in the response to comment #11 below).

Revision 1: *(line 147-152)* We applied random forest (RF, “randomForest” R package [77]) for regression because previous studies found that it was more robust to model population trends than regression-based or other machine-learning algorithms [78-79]. We then used the genetic algorithm (GA) of package “caret” for feature selection [80].

Revision 2: *(line 141-142)* The model equation is expressed as:

$$\lambda_{t}= \beta_{0}+\beta_{1}Y_{t-1}+\beta_{2}Y_{t-2}+\beta_{3}{X1}_{t}+\ldots+\beta_{17}{X15}_{t}+\varepsilon_{t}$$

Revision 3: *(line 143-146)* where λt is density growth rate from the previous generation to the current generation; Yt-1 and Yt-2 is the densities of one and two generations ago, respectively; Xnt are environmental factors of the current generation differing between the two generations. Since there were no “previous generations” for the first-year data, we started the models from the second year.

9. **Individual model fits (for generations and locations) prevent clear inference in terms of determining significant differences across space and between the two generations. One model that incorporates observations across all locations and generations would be much more useful. That is, stack up the response variables and predictor variables and then specify site specific and generation specific coefficients.**

Response 1: We had the models that incorporated all the locations of five populations together - the "All" models. However, this could largely dilute the resolution of our data, and was confirmed by our outputs (Table 2 & Table 3): for instance, it's common to see that the coefficient of a variable was positive in the All model while it became negative in the individual population models. These Kbb populations are highly isolated and may have made local adaptations. Therefore, the differences among populations were what we expected and having models at population scale could help us better capture the divergences. We actually used the All models as our reference (or "broad picture") to prove this.

Response 2: For the generation, as we explained in the response to comment #5, we still don't think combining two generations into one model is in the right direction. Again, these two generations are influenced by different factors (both density-dependent and -independent variables) because they are not occurring at the same time in a year and the second-generation density depends on the first generation, and thus they could not be easily stacked up. This is actually the core of this study and is also the reason why we called this as a "novel approach".

Revision: *(line 128-131)* We included both density-dependent and -independent variables to build endo-exogenous models specific to (i) each population, and (ii) all the surveyed locations of the five populations pooled together (All). We modeled the first and the second generation separately because they were mostly non-overlapped except in June and experienced different climate patterns [73-74].

10. **line 268: How do you assess which is more influential?**

Response: We thought you were asking about line 258. We used the estimated coefficient of PLS models to make this statement. The variable importance analysis on RF models also showed greater influences of density dependence on population growth rate, though they were not shown in the manuscript because it was highly consistent with PLS outputs.

Revision: *(line 214-215)* However, the density of the second generation in the previous year (PS) almost always had larger regression coefficients than the other density-dependent variable.

11. **How does a model that regresses Yt on Yt-1 lead to inference with regard to density dependence? Density dependence typically refers to a relationship between population growth rate and population density. If I'm understanding the model as written, you are actually just identifying positive autocorrelation.**

Response: We originally have models for both Yt and λt. The model performances of Yt were generally better than λt, so we included Yt in the previous manuscript. However, we realized that using Yt did not confer the conventional definition of density dependence and could rise the question on autocorrelation, we decided to switch to the models on λt.

Revision 1: *(line 100-101)* We also calculated the population growth rate (λ) based on density change between current and previous generations.

Revision 2: *(line 132-135)* Each model had λ as the response variable and 18 standardized explanatory variables: two previous-generation densities, four (IT, AT, MT, and PT) * three (egg, larvae/pupae, and flight) monthly climates, three topographies (elevation, slope, and trasp), and tree canopy.

12. **What is the model for Y1, i.e., how do you model the count data from year 1?**

Response: We did not model year 1; we started from year 2.

Revision: *(line 145-146)* Since there were no “previous generations” for the first-year data, we started the models from the second year.

# Reviewer 2:

1. **Line 30: For Whole Species the values are negative.**

Response: Yes, in the previous manuscript, Whole Species values were negative, which was the reason why we mentioned "generally". In the revised manuscript, since we decided to stick to modeling the growth rate of density instead of the density itself, the outputs were different from the previous version (see the Response to Comment #11 of Reviewer 1 for more details).

Revision: *(line 29-31)* The growth rate decreased with higher overwinter temperatures and spring precipitation in the first generation, while it increased with higher summer temperatures and precipitations in the second generation.

2. **Line 39: The insects are not only ectotherms. Many insects are capable of generating endothermic heat. Thus. the sentence is not correct (See Stone and Willmer, 1989 and others).**

Response: Yes, this is our mistake. We decided to remove the concept of ectotherms and focus on butterflies instead of insects in general.

Revision: *(line 35-37)* Butterflies, as good indicator species with rapid responses to fluctuations of abiotic conditions [1-6], are highly sensitive and vulnerable to climate change because many have reached the maximum adaptive capacity [7-8].

3. **Lines 40-41: What do you mean with: complex life cycles and varying vulnerability throughout their life spans?**

Response: This sentence could be confusing. Basically, different life stages in the life cycles of insects (now butterflies) have unique interactions with climate. We have made our expression clearer in the revised manuscript.

Revision: *(line 37-38)* Climate change interferes with butterflies by impacting development throughout the life cycle and changing interaction with host plants.

4. **Lines 44-45: this sentence is confusing.**

Response: The whole paragraph is more relevant to another manuscript of us where we used the models created in this manuscript to predict extinction risk and distribution on future climate change for Kbbs. So we decided to remove the whole paragraph.

Revision: *(previously line 43-49)* Removal of the whole paragraph.

5. **Line 47: italics for the species name. Important to add author and year of the species description.**

Response: The scientific name was changed to italic form. The author and the year of description were also added.

Revision: *(line 45-46)* The Karner blue butterfly (*Lycaeides melissa samuelis*, or Kbb), first described by Vladimir Nabokov in 1944, was listed as federally endangered in 1992 [23-25].

6. **Line 66: (Geiger et al., 2009) need a number.**

Response: The reference number should be ‘55’ and we forgot to convert it into this number. We also improved the sentence a little bit.

Revision: *(line 66-68)* For instance, south-facing slopes are generally warmer than north-facing slopes in the northern hemisphere; higher elevations are colder and drier than lower altitudes [55-56].

7. **Lines 69-71: the sentence requires reference.**

Response: We added back the reference numbers at the end of this sentence.

Revision: *(line 46-51)* Like many species in the family Lycaenidae, it is vulnerable to climate change because of limited dispersal ability (< 1 km), single host plant (i.e., monophagy on wild lupine, Lupinus perennis), and habitat specialist relying on high-quality oak savanna and pine barren which have been lost and fragmented for decades as the result of fire suppression, agricultural intensification, and urbanization [26-30].

8. **Line 70: how much would be the number of host plants to be considered limited?**

Response: we tried to make it clearer by using the phrase "monophagy" and we explicitly state "single host plant" in the revised sentence.

Revision: *(line 46-51)* Like many species in the family Lycaenidae, it is vulnerable to climate change because of limited dispersal ability (< 1 km), single host plant (i.e., monophagy on wild lupine, Lupinus perennis), and habitat specialist on high-quality oak savanna and pine barren which have been lost and fragmented for decades as the result of fire suppression, agricultural intensification, and urbanization [26-30].

9. **Line 71: The authors don't cite the IUCN Red List of Threatened Species status.**

Response: Has been added, and the reference number is 23.

Revision: *(line 45-46)* The Karner blue butterfly (*Lycaeides melissa samuelis*, or Kbb), first described by Vladimir Nabokov in 1944, was listed as federally endangered in 1992 [23-25].

10. **Lines 75-77: the sentence requires reference.**

Response: The sentence was incorporated into the same one responding to Comments #7 and #8.

Revision: *(line 46-51)* Like many species in the family Lycaenidae, it is vulnerable to climate change because of limited dispersal ability (< 1 km), single host plant (i.e., monophagy on wild lupine, Lupinus perennis), and habitat specialist on high-quality oak savanna and pine barren which have been lost and fragmented for decades as the result of fire suppression, agricultural intensification, and urbanization [26-30].

11. **Lines 97-101: this sentence is confusing.**

Response: The sentence was modified and embedded first paragraph.

Revision: *(line 40-44)* However, the impacts are mixed: it was found that warmer temperature facilitates egg and pupa survival, advances flight period, alters female fertility, and induces extra generations [13-15], while it decreases larva survival and adult body size [16-18]. Drought can reduce food availability and delay growth, but excessive rainfall may disrupt nectar and pollen foraging as well as egg laying [19-22].

12. **Lines 119-120: It is necessary to add more detail about sampling. E.g. the size of the transects, hours and days of sample activities.**

Response: More details about the sampling have been added to the text and new Table 1 (previously in SI).

Revision 1: *(line 86-93)* We used time-series data, of 8–27-year duration, from five Kbb populations across the species’ range (Table 1). Four populations were located on oak savannas in the Midwest U.S.: central Wisconsin (CW), northwest Wisconsin (NW), Fort McCoy (FM), and Indiana Dunes National Park (IDNP). One was from pine barrens in the eastern U.S.—Albany Pine Bush Preserve (APBP). Our data represent adult Kbbs surveyed at multiple locations (S1 Table) along one to several linear transects per location repeatedly every 7-14 day interval during the two flight periods (i.e., from late May to late June for the first generation, and from mid-July to mid-August for the second generation).

Revision 2: *(line 102-105)* Table 1.

13. **Lines 206-208: Add the statistical reasons found for the statement.**

Response: We added the p-value into the statement, though the negative associations were very straightforward based on Table 2 (previously Table 1).

Revision: *(line 173-175)* For the first generation, λ was negatively correlated with PS for All, CW, NW, and FM populations (p < 0.05) and with PF for IDNP and APB populations (p < 0.01, Table 2).

14. **Table 1: you put in the legend: Blank cells indicate not significant. However, there are p>0.1 values in the table.**

Response: In the new Table 2 (previously Table 1), we added in the legend b that "ns" indicates nonsignificant, and in the lengend c that blank cells represented exclusion from the best-fitted models.

Revision: *(line 184-185)* Table 2 legend b and c.

15. **Table 2: in the legend only: JN and in the table: JL and JN.**

Response: The explanation of JL has been added to legend e at the bottom of new Table 3 (previously Table 2).

Revision: *(line 208-210)* Table 3 legend e.

16. **Table 2: PF and in the table 1: CF. Are the same thing?**

Response: No, PF is the density of previous-year first generation and CF is the density of previous-year second generation. We revised the legends at the bottom of new Table 3.

Revision: *(line 208-210)* Table 3 legend e.

17. **Line 243: where are these values?**

Response: The adjusted-R^2^ are at the last row of the table. We indicated them in the title of Table 2 and 3.

Revision: *(line 182-183 & line 202-203)* The last row of Table 2 and 3.

# Editor

1. **Please ensure that your manuscript meets PLOS ONE's style requirements, including those for file naming.**

Response: We have modified all the documents to comply with the requirements.

2. **In your Methods section, please provide additional information regarding the permits you obtained for the work. Please ensure you have included the full name of the authority that approved the field site access and, if no permits were required, a brief statement explaining why.**

Response: The authority names were included.

3. **We note that Figure 1 in your submission contain [map/satellite] images which may be copyrighted. All PLOS content is published under the Creative Commons Attribution License (CC BY 4.0), which means that the manuscript, images, and Supporting Information files will be freely available online, and any third party is permitted to access, download, copy, distribute, and use these materials in any way, even commercially, with proper attribution. For these reasons, we cannot publish previously copyrighted maps or satellite images created using proprietary data, such as Google software (Google Maps, Street View, and Earth). For more information, see our copyright guidelines:** [**http://journals.plos.org/plosone/s/licenses-and-copyright**](http://journals.plos.org/plosone/s/licenses-and-copyright)**. We require you to either (1) present written permission from the copyright holder to publish these figures specifically under the CC BY 4.0 license, or (2) remove the figures from your submission.**

Response: We decided to remove Fig 1.
