# Supplementary material for: Extinction risk modeling predicts range-wide differences of climate change impact on Karner blue butterfly (Lycaeides melissa samuelis)
Source: PLoS One. 2023 Nov 7;18(11):e0262382. doi: 10.1371/journal.pone.0262382 (PMC10629659; doi:10.1371/journal.pone.0262382)
Supplement: Attachment — Submitted filename: Response to Reviewers.docx [file pone.0262382.s007.docx]

# Reviewer 3:

1. **This revised version is ok, apart from one thing I apparently did not notice during the previous readings of the manuscript. When reading it now, I find that there are a lot of abbreviations that might be familiar to the authors, but not to an international readership. I give a (somewhat exaggerated and fictious) example to make my case: “The λ of the Kbb in the IDNP and APBP populations in the NW was mostly determined by PF and not by AT, MT or PT resulting form the GA approach” … :-D As a reviewer I have to read the whole manuscript and I try to remember as many abbreviations as possible (NOT!), but as a scientist/reader, I usually only read parts of a paper (last paragraph of the introduction, conclusion, figures …). When I come across sentences like the one I wrote, I usually stop reading because I have to struggle to hard to find out what all the abbreviations mean and that would be a pity for this very interesting work! In the era of printed versions, I can understand that limiting the number of characters was important, but with (almost) everything being digital, the use of full words instead of abbreviations would make it much more reader-friendly. If the editor of PONE is ok with abbreviations, fine by me, but I don’t like it …**

Response: We went through the Methods, Results, and Discussion sections and changed all the abbreviations that may cause confusions.

Revision: For instance, (*line 176 – 177*) the two abbreviated population names IDNP and APBP were revised to Indiana Dunes National Park and Albany Pine Bush Preserve for clarification.

2. **A simple figure with a map where the study sites are located, together with the abbreviations if you want, would make localising the sites at least a lot easier (not everyone is American and knows where all American states are situated).**

Response: We added a muti-paneled figure mapping out the locations of all the permanent sampled sites of the five populations of Karner blue butterfly.

Revision: (*line 93 – 97*) Captions of Fig 1.

3. **The potential additional reference that could be included in the section about climate warming leading to extra generations and the possible negative consequences of it is “Van Dyck H, Puls R, Bonte D, Gotthard K & Maes D (2015) The lost generation hypothesis: could climate change drive ectotherms into a developmental trap? Oikos 124 (1): 54-61. https://doi.org/10.1111/oik.02066.” but do not feel obliged to include it by any means.**

Response: We thought this potential refence was relevant to our manuscript, as we did briefly mention the potential of a third generation. We therefore still decided to add this reference and write out a new sentence about the caveat due to the “developmental trap” demonstrated by Van Dyck et al. (2015).

Revision: (*line 257 – 258*) However, the envisaged third generation, if present, worth further research as this could be a maladaptive response if novel conditions in fall were unfavorable [111].
